# Supplementary material for: Penile-Sparing Surgery for Tumour Recurrence after Previous Glansectomy/Partial Penectomy: Treatment Feasibility and Oncological Outcomes
Source: Cancers (Basel). 2023 Sep 29;15(19):4807. doi: 10.3390/cancers15194807 (PMC10571586; doi:10.3390/cancers15194807)
Supplement: Supplementary file 1 [file cancers-15-04807-s001.zip › Figure S1.pdf]

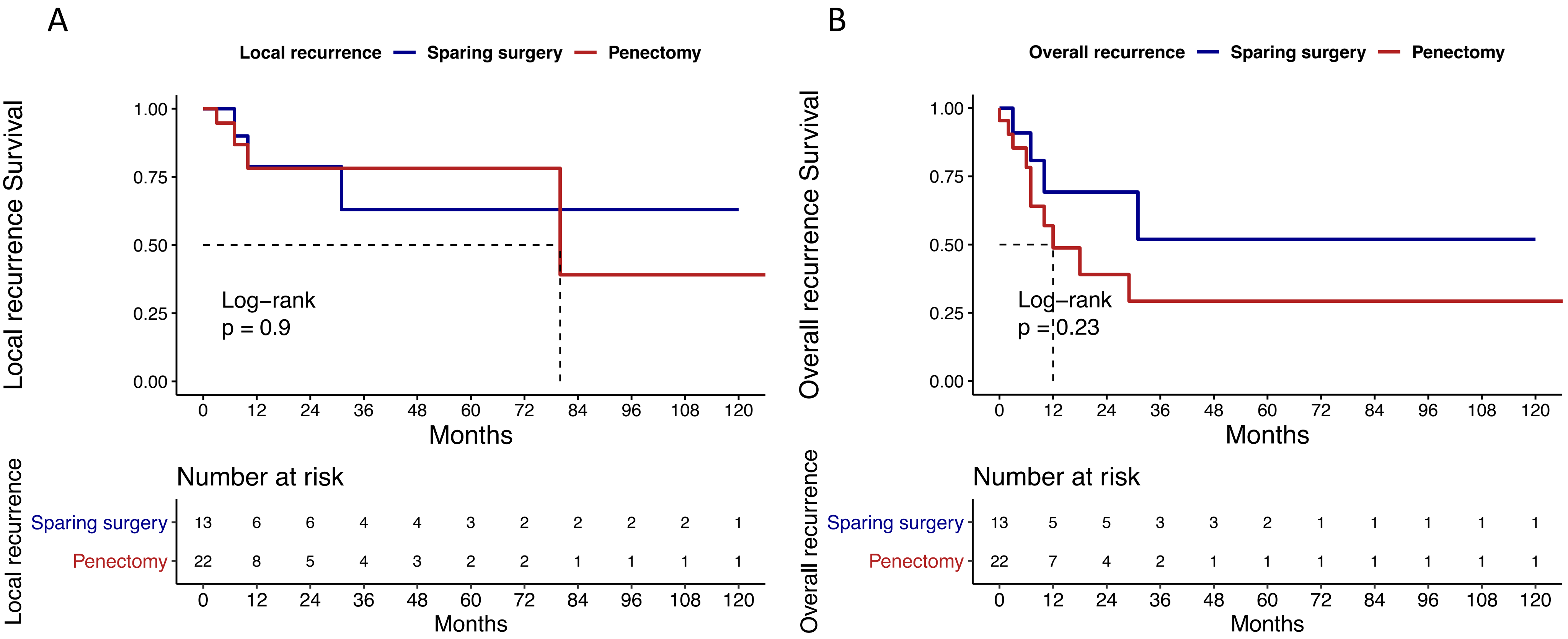
A
Local recurrence
Sparing surgery
Penectomy
Local recurrence Survival
1.00
0.75
0.50
0.25
0.00
Log−rank
p = 0.9
0 12 24 36 48 60 72 84 96 108 120
Months
Number at risk
Local recurrence
Sparing surgery 13 6 6 4 4 3 2 2 2 2 1
Penectomy 22 8 5 4 3 2 2 1 1 1 1
0 12 24 36 48 60 72 84 96 108 120
Months
B
Overall recurrence
Sparing surgery
Penectomy
Overall recurrence Survival
1.00
0.75
0.50
0.25
0.00
Log−rank
p = 0.23
0 12 24 36 48 60 72 84 96 108 120
Months
Number at risk
Overall recurrence
Sparing surgery 13 5 5 3 3 2 1 1 1 1 1
Penectomy 22 7 4 2 1 1 1 1 1 1 1
0 12 24 36 48 60 72 84 96 108 120
Months
