# Supplementary material for: Penile-Sparing Surgery for Tumour Recurrence after Previous Glansectomy/Partial Penectomy: Treatment Feasibility and Oncological Outcomes
Source: Cancers (Basel). 2023 Sep 29;15(19):4807. doi: 10.3390/cancers15194807 (PMC10571586; doi:10.3390/cancers15194807)
Supplement: Supplementary file 1 [file cancers-15-04807-s001.zip › Table S1.pdf]

Table S1. Clinical characteristics of 22 penile cancer patients, previously treated with glansectomy or partial penectomy, and subsequently treated radical penectomy for penile cancer recurrence between 2001 and 2022. Data are shown as medians for continuous variables or as counts and percentages (%) for categorical variables.

**Age (years)**	Median (IQR)	61 (51-69)
**CCI, n (%)**	1	7 (32.0)
	2	6 (27.0)
	≥ 3	9 (41.0)
**Previous surgery, n (%)**	Partial/total glansectomy	15 (68.0)
	Partial penectomy	7 (32.0)
**Previous histology, n (%)**	Squamous cell	20 (91.0)
	Epidermoid	1 (4.5)
	Unknown	1 (4.5)
**Previous T stage, n (%)**	Unknown	2 (9.0)
	T1	3 (13.5)
	T2	9 (41.0)
	T3	8 (36.5)
**Previous tumor grade, n (%)**	Gx	4 (18.0)
	G1	3 (13.5)
	G2	5 (22.5)
	G3	10 (46.0)
**Previous N stage, n (%)**	Nx	5 (22.5)
	N0	14 (64.0)
	N1	3 (13.5)
**Time to recurrence (months)**	Median (IQR)	11 (5-63)
**Tumor size (mm)**	Median (IQR)	27.5 (20-47.5)
**Histology**	Squamous cell	17 (77.0)
	Verrucous	1 (5.0)
	Unknown	2 (9.0)
	Negative	2 (9.0)
**T stage, n (%)**	Unknown	4 (18.0)
	Ta	1 (5.0)
	T1	2 (9.0)
	T2	4 (18.0)
	T3	7 (32.0)
	PeIN	2 (9.0)
	Negative	2 (9.0)

IQR: interquartile range
